# First isolation of *Klebsiella variicola* from a horse pleural effusion

**DOI:** 10.1186/s12917-021-02776-2

**Published:** 2021-02-12

**Authors:** Elisabetta Mondo, Riccardo Rinnovati, Alessandro Spadari, Federica Giacometti, Andrea Serraino, Federica Savini, Silvia Piva

**Affiliations:** Department of Veterinary Medical Sciences, University of Bologna, Via Tolara di Sopra 50, Bologna, 40064 Italy

**Keywords:** *Klebsiella variicola*, Horse, Respiratory disease

## Abstract

**Background:**

Respiratory diseases are the second most common cause of illnesses in horses, their etiology can be viral, bacterial, immune-mediated, or mechanical (Racklyeft and Love DN, Aust Vet J 78:549–59, 2000; Austin et al., J Am Vet Med Assoc 207:325–328, 1995; Arroyo et al., J Vet Intern Med 31:894–900, 2017). *Klebsiella variicola* is a Gram-negative bacterium that was initially identified as an endophyte in soil and plants such as bananas, rice, sugar cane and maize but recent studies have identified this microorganism as an emerging pathogen in humans (Rodríguez-Medina et al., Emerg Microbes Infect 8:973–988, 2019; Fontana et al., J Clin Microbiol 57:e00825–18, 2019; Rosenblueth et al., Syst Appl Microbiol 27:27–35, 2004).

This paper describes, for the first time to our knowledge, the isolation of K. variicola from pleural effusion in a male adult horse.

**Case presentation:**

17-years Italian Saddle Horse with respiratory distress and fever was admitted to the Veterinary Teaching Hospital of the Department of Veterinary Medical Sciences, University of Bologna. At home, the patient had undergone antibiotic therapy without clinical improvement. Vital signs on admission revealed an increased respiratory rate, tachycardia, pyrexia and weight loss. The animal was submitted for collateral examination including thoracic radiology and ultrasound and thoracoscopy that showed bilateral pleural effusion associated with multifocal pulmonary atelectasis.

During the thoracoscopic examination, that confirmed the presence of a seropurulent pleural effusion, a sample of pleural fluid was collected and Gram-negative bacteria were isolated and subjected to matrix-assisted laser desorption/ionization time-of-flight mass spectrometry (MALDI-TOF MS) that allowed the identification of *K. variicola*. The isolate was sensitive to amikacin, cefazolin, enrofloxacin, marbofloxacin, tetracycline, and trimethoprim-sulfamethoxazole;the horse was treated with Oxytetracycline and amikacin. Despite a general health improvement of the subject, the pleural effusion did not resolve after treatment.

**Conclusions:**

This paper describes, for the first time, the isolation of *K. variicola* in a horse with respiratory disease. The misidentification between *K. variicola* and *K. pneumoniae* has caused unawareness about significant aspects of this bacterial species. In fact, even though in animals the role of this bacterium is not clear, in humans it has been recognized as an emerging pathogen. The use of new methods for bacterial identification will probably lead to the isolation of a greater number of strains which will have to be studied to acquire knowledge that will be useful to clarify the clinical importance and relevance of *K. variicola* also in animals.

## Background

Respiratory diseases are the second most common cause of illnesses in horses, the etiology can be viral, bacterial, immune-mediated, or mechanical [1]. Pleuropneumonia is not uncommon in horses, the most common risk factors include excessive exercise, transportation and viral respiratory infections that lead to an immune suppression status [2]. *Streptococcus equi* subsp. *zooepidemicus* is the most common bacteria isolated from horses with pleuropneumonia, but many other opportunistic pathogens have been isolated in cases of pneumonia, also often caused by mixed infections between aerobic and anaerobic bacteria. Other common aerobic bacteria isolated from pleuropneumonia include *Streptococcus* spp., *Pasteurella* spp., *Escherichia coli*, *Klebsiella* spp. *Actinobacillus* spp., *Enterobacter* spp.; *Bacteroides* spp., *Clostridium* spp., *Fusobacterium* spp. and *Peptostreptococcus* spp. as anaerobes [3].

*Klebsiella variicola* is a Gram-negative, nitrogen-fixing, nonspore-forming, nonmotile rod-shaped bacterium, firstly isolated in Mexico in 2004 and identified on the basis of total DNA-DNA hybridization and phylogenetic analysis of the sequences of *rpoB* gene [4]. *K. variicola* was initially identified as an endophyte in soil and plants such as bananas, rice, sugar cane and maize, but recent studies identify this microorganisms as an emerging pathogen in humans; it has been isolated from many clinical samples, including blood, tracheal aspirates, several types of secretions as well as the respiratory and urinary tract [4–7]. In animals, *Klebsiella* spp. are associated with infections of the urinary tract, respiratory tract and sepsis [8], while *K. variicola* has to date only been described in a bovine mastitis [9].

This paper describes, for the first time to our knowledge, the isolation of *K. variicola* from pleural effusion in a male adult horse showing clinical signs associated with respiratory disease, highlighting the risk of misidentification with *Klebsiella pneumoniae*.

## Case presentation

**Fig. 1:**
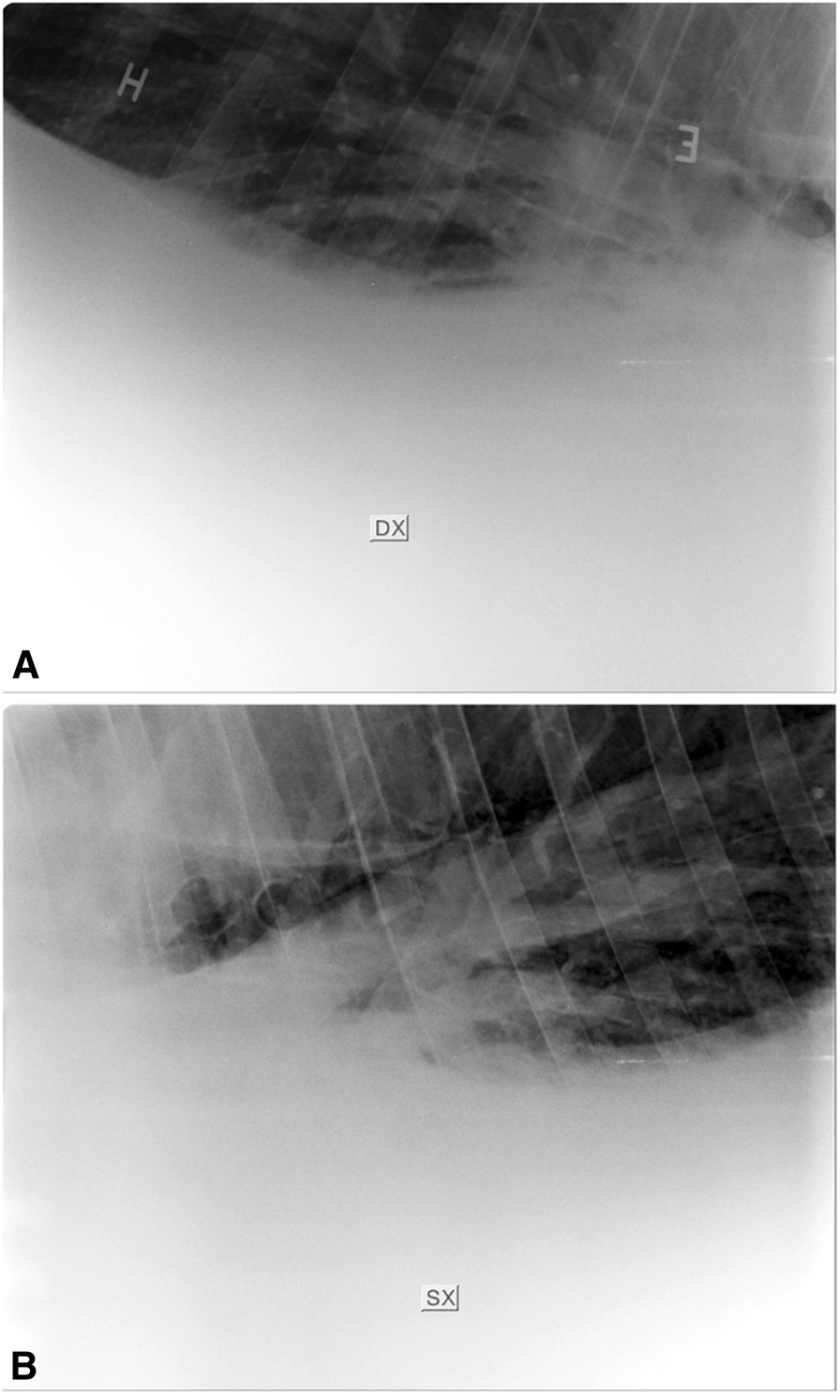
LL views Right to Left (**a**) and Left to Right (**b**). The radiograms show the hydrollevel

**Fig. 2:**
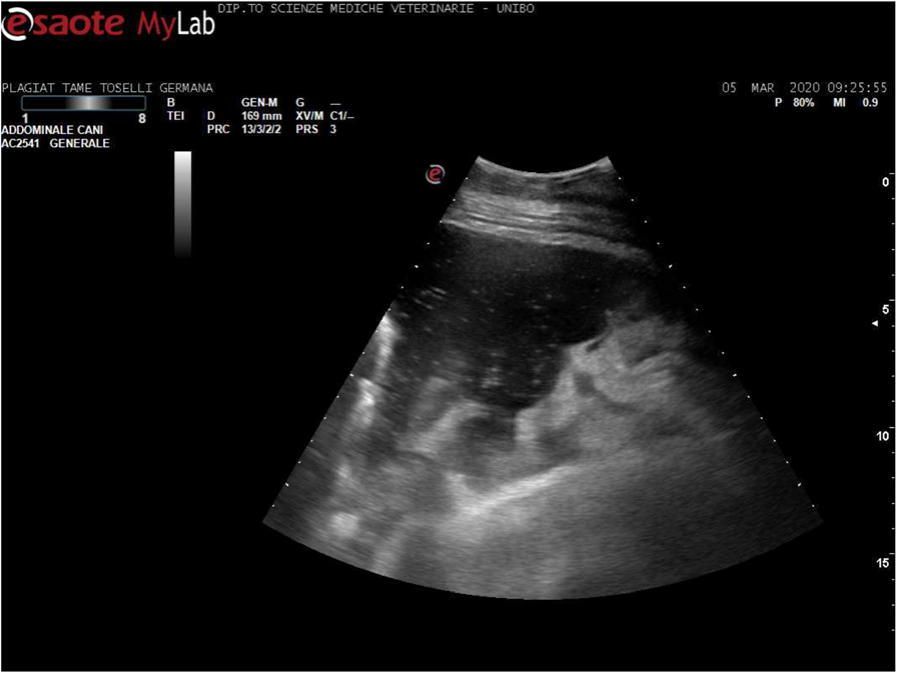
Ultrasound image of pleural space with pleural effusion and pleural fluid

In February 2020, a 17-years Italian Saddle Horse male with respiratory distress and fever was admitted to the Veterinary Teaching Hospital of the Department of Veterinary Medical Sciences, University of Bologna. At home, the patient had undergone antibiotic therapy in a sequence (Penicillin G- procain 44,000 IU/KG for five days every 24 h IM; Oxitetracicline 6.6 mg/kg for 5 days every 24 h IV; Ceftiofur sodium 2.2 mg/kg IV every 12 h for ten days), without clinical improvement. Vital signs on admission revealed an increased respiratory rate (> 15 breaths/min), tachycardia (heart rate > 44 beats/min; 56/64), pyrexia (> 38.5 °C; 31/62), and weight loss (15/59). Further collateral examination including thoracic radiology and ultrasound and thoracoscopy, were performed. The radiographic examination, performed in three left to right to left latero-lateral views (Fig. 1), revealed a bilateral line of fluid extended across the entire chest, with an approximate height of 20 cm on the right side and 17 cm on the left side, in particular at the mediastinal level. The ultrasound examination showed bilateral pleural effusion associated with multifocal lung atelectasis. The latter was more severe in the right hemithorax where the parietal and diaphragmatic pleura were also involved (Fig. 2). The thoracoscopic examination confirmed the presence of a seropurulent pleural effusion, but no superficial obvious lesions to the lung and to the parietal and visceral pleura were evidenced. Along with the first trocar insertion, a sterile sample of turbid yellow thoracic fluid was collected and sent to the laboratory for cytological and bacteriological exams. Cell count was performed with ADVIA 2120 and resulted in 43’000 eritrocytes/µl, 91’267 total nucleated cells /µl with 92 % non-degenerated or picnotic neutropils, 4 % activated macrophages and 4 % small lymphocytes. Neither intra nor extracellular microorganisms were observed.

The haematology did not reveal anything particularly significant.

Under thoracoscopic guidance a second opening for drainage tube insertion was created at the 9th right intercostal space 10 cm dorsal to a horizontal line passing by the olecranic tuberosity. A drainage tube of 28 F was inserted for daily evacuation of the fluid and pleural wash.

**Fig. 3:**
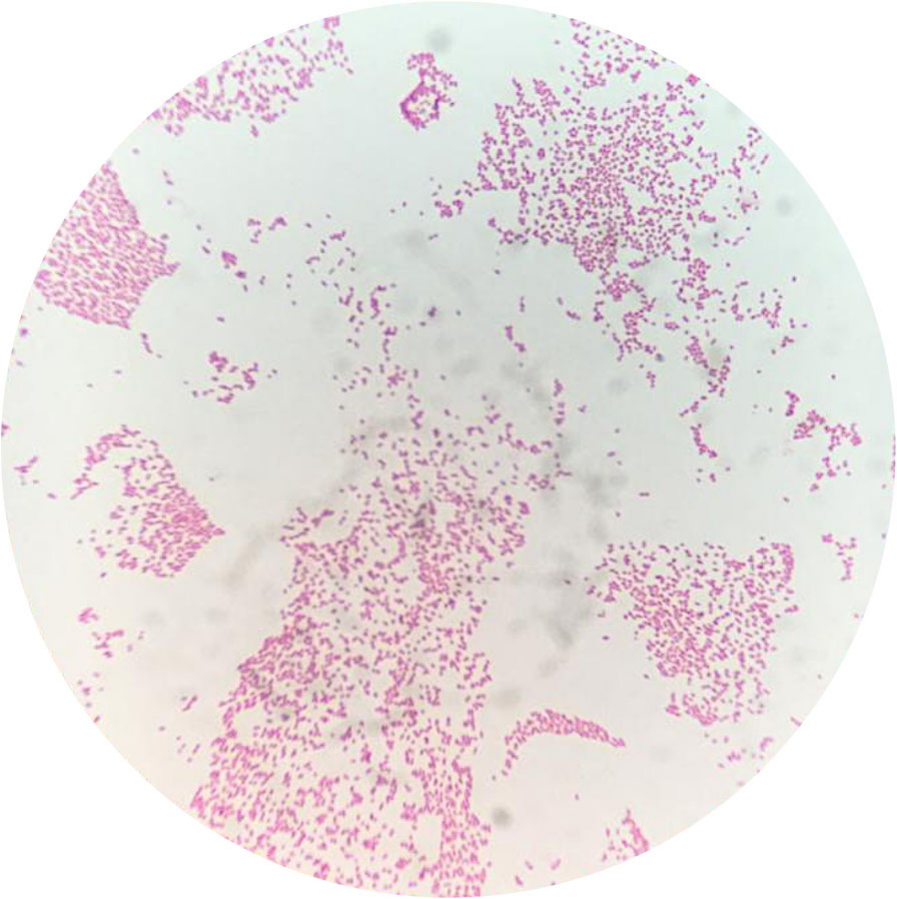
Gram stain of *Klebsiella variicola*. The Gram stain of a positive culture demonstrates Gram-negative bacilli on microscopy (x100)

A sample of pleural fluid collected during thoracoscopy was collected for routine aerobic and anaerobic bacterial culture. After 24 h of incubation, round and mucoid colonies were isolated on Blood Agar Base with 5 % horse blood and on MacConkey agar plates. Microscopically, colonies appeared as Gram-negative rod-shaped (Fig. 3) and, by biochemical tests, they were catalase-positive and oxidase-negative. An isolate from Blood Agar was subcultured on Tryptone Soya Agar and incubated for 24 h at 37 ± 1 °C in aerobic condition. Subsequent identification was performed using matrix-assisted laser desorption/ionization time-of-flight mass spectrometry (MALDI-TOF MS) (MALDI biotyper, Bruker Inc., USA) instrument: *K. variicola* was identified at species level with a score of 2,25 using the BRUKER BIOTYPER version 3.0 software. An antimicrobial susceptibility test was performed by means of the disc diffusion method according to Clinical and Laboratory Standards Institute (CLSI) guidelines [10, 11] by using antimicrobial discs. The isolate was sensitive to amikacin (30 µg), amoxicillin- clavulanate (30 µg), chloramphenicol (30 µg), cefazolin (30 µg), enrofloxacin (5 µg), marbofloxacin (5 µg), tetracycline (30 µg), and trimethoprim-sulfamethoxazole (1.25/23.7 µg), Intermediate sensitivity was evidenced to gentamicin (10 µg), streptomycin (10 µg), ceftiofur (30 µg), while the isolates was resistant to ampicillin (10 µg), and sulfonamides (300 µg).

A combination disc test following the instructions reported in EUCAST (2017) for the evaluation of ESBL was performed (Oxoid, Basinstoke, UK) media and antimicrobial discs. The isolate was tested for ceftazidime alone and in combination with clavulanic acid using Kirby Bauer disk agar diffusion method [12].

The inhibition zones of the cephalosporins alone, as well as in combination with clavulanic acid were compared. The test was considered positive when an increase ≥ 5 mm in zone of inhibition was observed in the presence of clavulanic acid, compared with the cephalosporin alone but in our isolate the ≥ 5 mm increase was not observed. Oxoid (Basinstoke, UK) media and antimicrobial discs were used.

According to the results, the horse was treated I.V. with Oxytetracycline (6,6 mg/kg sid) for 10 consecutive days. In addition, a daily intrathoracic lavage with amikacin (400 gr) diluted in 2 liters of saline was administered. The general health of the horse improved after 10 weeks from hospital discharge but the pleural effusion was not resolved.

## Discussion and conclusions

In this paper, isolation of *K. variicola* from pleural effusion in a male adult horse with respiratory symptoms is reported. Although a single isolation from a unique pleural effusion sample has been performed, *K. variicola* grew in a monomicrobic growth. In humans, *K. variicola* is considered an emerging pathogen, while in animals its role is still unclear. The study carried out by Maatallah et al. (2014) reported that human bacteremia infections caused by *K. variicola* have higher 30-day mortality rates than those of infections caused by *K. pneumoniae*, suggesting the higher virulence of *K. variicola* [13]. Moreover, several researches estimate that around 10 % of *K. pneumoniae* isolated in human infections are misidentifications of *K. variicola* [6]. In literature, *K. variicola* has rarely been isolated in animals [8], in particular, among food-producing animals Podder and Colleagues (2014) evidenced that an isolate of *K. variicola* can cause clinical mastisis in dairy cattle, as it is normally found in soil and feed, and not in milk from infected animals [9].

Biochemical and phenotypic features’ overlapping between *K. pneumoniae* and *K. variicola* make identification of the latter difficult by traditional microbiological methods [14], leading to underestimate its pathological importance.

Furthermore, since the sequences of *K. variicola*, and *K. quasipneumoniae*, have been included in common molecular databases only recently, the two species have been either unidentified or misidentified. Misidentification has caused unawareness about significant aspects of this bacterial species, that is becoming a public health concern not only for the infections that it can cause but also due to its potential to acquire antimicrobial and virulence genes, hampering the clinical management of the provoked infections [15].

The available methods differentiating and identifying bacterial subspecies within the *K. pneumoniae* complex, such as *K. variicola*, were only based on genomic and phylogenetic analysis, until a few years ago. In particular, genomic analysis is based on PCR amplification of chromosomal β-lactamases, multiplex PCR, or PCR for the research of *yggE* gene, while phylogenetic analysis is based on the most commonly used gene *rpoB* [4, 16].

In 2018, *K. variicola* was added to the MALDI-TOF MS Bruker reference library, and shortly after a study demonstrated 100 % sensitivity and specificity [14] of this methodology in differentiating among *K. pneumoniae, K. quasipneumoniae*, and *K. variicola*. Hence, the use of MALDI- TOF for bacterial identification in routine microbiology is preferable to conventional phenotypic/biochemical techniques in terms of speed and precision. Nevertheless, PCR and phylogenetic analysis represent a valid alternative when MALDI-TOF technology is not available.

Although successfully applied for the identification of human pathogens, so far its use for routine identification of veterinary bacterial isolate is still limited [17].

Correct identification of *K. variicola* is of the utmost importance because this new bacterium may be a pathogen agent in the animals like it is classified as an important human pathogen. Correct identification of the respiratory infection causative agent is the first step for the right administration of a specific antimicrobial therapy, and it is fundamental for the animal’s healing and the prudent use of antibiotics. In line with our findings, *K. variicola* isolates are broadly antimicrobial susceptible (even if reports of ESBL-producing and carbapenemase-producing isolates have increased) [5] and have lower antibiotic resistance rates than other *Klebsiella* species. Nevertheless lower antibiotic resistance rates do not necessarily correlate with better treatment outcomes in *K. variicola* infections [18]. In addition, the genome of *K. variicola* is considered an open genome, namely, the microorganism is able to incorporate genes that allow its adaptation to different environments by conferring resistance to antibiotics to which it was previously susceptible, as well as expanding its pathogenicity by incorporating virulence factors [19].

Further efforts should be performed to differentiate *K. variicola* from *K. pneumoniae* complex since adequate identification of *K. variicola* is not routinely performed in clinical specimens and its real incidence is unknown. The use of new methods for bacterial identification will probably lead to the isolation of a greater number of strains that will widen knowledge on thei pathogenic power and diffusion, as well as on the clinical importance and relevance of *K. variicola* in human and animal infection.

### Conclusion

The present study describes the isolation of *K. variicola* in a horse with respiratory disease. In the past, misidentification with *K. pneumoniae* has caused unawareness about significant aspects of this bacterial species, as for example its possible role in animals’ diseases. In fact, probably due to the poor isolation rate its role is not clear in animals, while it has been demonstrated, to be an emerging human pathogen. Further studies will be needed to understand the spread and the virulence pattern of *K. variicola* in veterinary medicine.

## Data Availability

The datasets used and/or analyzed during the current study are available from the corresponding author on reasonable request.

## Abbreviations

MALDI-TOF MS: Matrix-assisted laser desorption/ionization time-of-flight mass spectrometry
CLSI: Clinical and Laboratory Standards Institute
EUCAST: European Committee on Antimicrobial Susceptibility Testing
ESBL: Extended Spectrum-Beta-Lactamase
